# Modulation of brain alpha rhythm and heart rate variability by attention-related mechanisms

**DOI:** 10.3934/Neuroscience.2019.1.1

**Published:** 2019-03-04

**Authors:** Elisa Magosso, Giulia Ricci, Mauro Ursino

**Affiliations:** Department of Electrical, Electronic and Information Engineering, Campus of Cesena, University of Bologna, Cesena (FC), Italy

**Keywords:** electroencephalography, alpha power, attention, Independent Component Analysis, Heart Rate Variability

## Abstract

According to recent evidence, oscillations in the alpha-band (8–14 Hz) play an active role in attention via allocation of cortical resources: decrease in alpha activity enhances neural processes in task-relevant regions, while increase in alpha activity reduces processing in task-irrelevant regions. Here, we analyzed changes in alpha-band power of 13-channel electroencephalogram (EEG) acquired from 30 subjects while performing four tasks that differently engaged visual, computational and motor attentional components. The complete (visual + computational + motor) task required to read and solve an arithmetical operation and provide a motor response; three simplified tasks involved a subset of these components (visual + computational task, visual task, motor task). Task-related changes in alpha power were quantified by aggregating electrodes into two main regions (fronto-central and parieto-occipital), to test regional specificity of alpha modulation depending on the involved attentional aspects. Independent Component Analysis (ICA) was applied to discover the main independent processes accounting for alpha power over the two scalp regions. Furthermore, we performed analysis of Heart Rate Variability (HRV) from one electrocardiogram signal acquired simultaneously with EEG, to test autonomic reaction to attentional loads. Results showed that alpha power modulation over the two scalp regions not only reflected the number of involved attentional components (the larger their number the larger the alpha power suppression) but was also fine-tuned by the nature of the recruited mechanisms (visual, computational, motor) relative to the functional specification of the regions. ICA revealed topologically dissimilar and differently attention-regulated processes of alpha power over the two regions. HRV indexes were less sensitive to different attentional aspects compared to alpha power, with vagal activity index presenting larger changes. This study contributes to improve our understanding of the electroencephalographic and autonomic correlates of attention and may have practical implications in neurofeedback, brain-computer interfaces, neuroergonomics as well as in clinical practice and neuroscience research exploring attention-deficit disorders.

## 1. Introduction

Neural oscillations between 8–14 Hz (*alpha frequency band*) are the prevalent oscillations in the human waking brain and ubiquitous throughout the cortex [1]. In the electroencephalographic recordings (EEG) at the scalp, the largest alpha amplitude is observed over parietal and occipital regions, but alpha rhythm is also evident over frontal and central sensorimotor regions (also called Rolandic alpha or mu rhythm) [1].

For a long period, alpha activity has been interpreted to reflect default states of cortical “idling” [2]. This interpretation was based on the classical observations that large alpha amplitude is characteristic of resting or deactivated cortical areas (in which no information is currently processed), while alpha is blocked by cortical activation due to sensory and/or motor processing.

Contrary to this earlier interpretation of alpha rhythm as a generic “idling” mechanism, evidence has been accumulated in recent years supporting a functional active role of alpha oscillations in information processing and task execution. A strong piece of evidence comes from the observations that alpha activity can exhibit either a decrease (alpha desynchronization) or an increase (alpha synchronization) in amplitude/power in response to a stimulus or task demand [1]. In particular, recent theories postulate that alpha-band oscillations act as a top-down inhibitory mechanism, implicated in attention [3],[4]. According to this hypothesis, an increase in alpha amplitude/power reduces information processing in the underlying neural networks; on the other hand, a decrease in alpha amplitude/power enhances information processing in the corresponding circuits. This hypothesis is supported by several studies covering a broad range of different tasks. For instance, a large amount of studies have investigated alpha power modulation in spatial cuing tasks and feature-detection tasks (for a review see [5],[6]). Alpha power suppression is larger over regions encoding the task-relevant feature, while alpha power increases over regions processing distracting or interfering features that need to be actively ignored. In other studies, alpha power modulation has been analyzed in the context of problem solving and arithmetic computation [7]–[9]. Results show that alpha power decreases when subjects become engaged in cognitive demanding tasks, alpha band suppression becoming larger as task complexity increases. Studies investigating working memory, long-term memory, and creative thinking [10]–[12] (for a compendium also see [6]) show that posterior alpha power increases in time periods related to retention and/or manipulation of internally represented information, suggesting inhibition of interfering external inputs.

In sum, modulations of alpha power are evident as power suppression in regions that are required for task execution (regions whose responsiveness must be enhanced), and as power increase in regions where distracting information has to be suppressed (regions whose receptivity must be inhibited). Hence, alpha activity, by selectively modulating the level of excitation/inhibition of cortical regions, has the function of enhancing neural processes within the task goals and blocking processes outside these goals. Such function can be considered as the operative definition of *top-down attention*. Indeed, top-down attention can be defined as the mechanism that prioritizes accessing to and processing of that subset of information deemed to be of the highest relevance for successful task completion [6]. Here we refers to attention as this endogenous (top-down) form of attention (i.e. under executive control), which is distinct from exogenous attention captured in an involuntary fashion. Accordingly, oscillatory alpha activity appears to support top-down attentional processes, its selective modulation shaping the functional architecture of the brain depending on the task at hand [3]–[6].

The aim of the present work is to contribute to this field by investigating the power changes in on-going alpha activity during tasks that differently engage distinct forms of attention. To this aim, we quantified EEG alpha power during a complete mathematical task involving a visual attentional component (reading numbers and symbols of an arithmetical operation on a computer screen), a computational component (mentally solving the arithmetical operation), and a motor component (selection and pushing of a button). Then, in order to evaluate the impact of each attentional component, we asked the subjects to perform also some simplified tasks decomposing the complete task in its subcomponents (a visual + computational task, a motor task, a visual task); alpha power changes in the simplified tasks were compared with those in the complete task (visual + computational + motor). We posit that this study, by analyzing the relative role of each attentional component to a composite task, may provide a novel contribution to further elucidate the relationship between attention and alpha-band activity. Indeed, our design is at variance with most studies on alpha power in cognitive tasks that often compare different levels of complexity of the same task [8],[13]–[15] or different strategies used for solving the task [7],[15], while only few treated decomposition of a composite task into its attentional subcomponents [16]. In particular, based on the hypothesis that alpha suppression is a mechanism that selectively gates cortical information processing and transfer, we investigated two main problems within this study. First, we analyzed the relationship between alpha power and the involved attentional components. In particular, we expect that the larger the number of attentional components (requiring larger excitation and coordination across cortical areas) the lower the alpha power. Moreover, we investigated whether the relationship between alpha power and the four different tasks is the same across the scalp or is modulated differently over different regions, depending on the specific nature of the involved attentional components. We expected a regional specificity of task-related alpha power modulation, supporting the hypothesis that the involved attentional components elicit a differential participation and activation of distinct brain areas. These issues were unraveled by aggregating electrode sites into two main regions of interest for alpha power measurement: parieto-occipital region and fronto-central region. Second, using Independent Component Analysis (ICA), we investigated whether individual brain processes, more specifically linked to visual-computational or to motor aspects of attention, can be extracted from a low-resolution EEG, for supporting the previous results and for future possible use in neuroengineering tools. Indeed, the possibility of extracting relevant features from low-resolution EEG is becoming increasingly attractive especially to realize easy-to-use, comfortable, and cheaper systems to be used in practical applications such as neurofeedback, brain computer interfaces, neuroergonomics, neuromarketing [17]–[19].

As a further point of novelty, in this study we acquired both EEG and ECG during each task and we performed analysis of Heart Rate Variability (HRV), besides quantification of alpha power. As well known, HRV refers to the physiological fluctuations in the heart period reflecting control of the cardiac activity via the sympathetic and parasympathetic branches of the Autonomic Nervous System (ANS). The power spectral density of the HRV presents two main frequency bands of functional interest: the Low Frequency band (LF, 0.04 to 0.15 Hz) interpretable as an index of the sympathetic activity and the High Frequency band (HF, 0.15 to 0.4 Hz) mainly reflecting the parasympathetic component. The LF/HF power ratio is considered a marker of the sympathovagal balance. HRV indexes have been shown to be modulated under tasks requiring attention (see for example [9],[13],[20]). Thus, as a further purpose of this study we also tested whether the indexes of HRV were sensitive to the engagement of different attentional components and we examined potential correlations between HRV changes and alpha power changes.

## 2. Materials and methods

### 2.1. Participants

Thirty healthy subjects (10 females) voluntarily participated in the study. They were recruited among students and employees of the University of Bologna (Italy). Their ages ranged from 20 to 42. Each participant had normal or corrected to normal vision and reported no medical or psychiatric illness. The study was approved by the local ethical committee and all participants gave written informed consent before the beginning of the experiment. All data were analyzed and reported anonymously.

### 2.2. Experimental protocol

The participant comfortably seated facing a computer monitor at about 50 cm far, in a quite laboratory. They underwent four consecutive experimental sessions, each lasting 15 minutes. Each single experimental session consisted of three phases: a 5-min initial relaxation phase (named R1), a 5-min central task phase (named T), a 5-min final relaxation phase (named R2). The four experimental sessions differed only in the type of the task executed during the central phase; the tasks were designed to include a complete task involving a set of attentional components and three other tasks involving a subset of these components.

**Figure 1.**
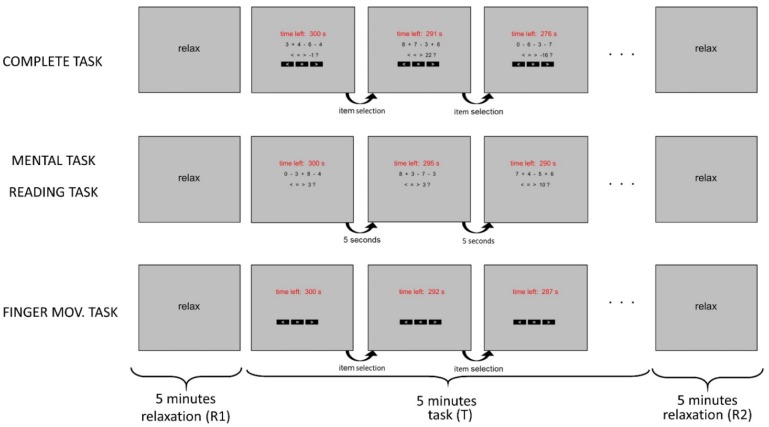
Diagram of the structure of each of the four experimental sessions. Each experimental session included an initial relaxation phase (R1) lasting 5 minutes, a central task (T) lasting 5 minutes, and a final relaxation phase (R2) lasting 5 minutes. Each experimental session differed only for the central task. In the complete task, inequalities appeared on the monitor and the participant had to solve the inequalities (first computing the result of the operation) and select the correct item (one of the black boxes with symbols < = >) with the mouse; after selection, a new inequality appeared. In the mental task, the participant had to solve the inequalities mentally without providing any explicit response. In the reading task, the participant had only to mentally read the numbers appearing on the monitor, without performing any computation. In both the reading and mental tasks, new appearances succeeded every 5 seconds. In the finger movement task, the participant had to randomly choose one selection item, using the mouse (about every 5–10 seconds) and no arithmetic operation was displayed. In each task, the time left within the task phase appeared on the screen. During the relaxation phases, a grey screen with the word ‘RELAX’ was displayed continuously while participant had to relax.

The complete task (Figure 1) consisted in solving inequalities presented on the screen and in providing the response using the mouse. In order to solve the inequality, the participants had to compute the result of an arithmetic operation consisting in the addition/subtraction of four one-digit numbers, and then compare the result with the provided target. They indicated their decision by selecting the appropriate item on the screen (one of the black boxes with the symbols <, =, >), by moving the mouse and pressing the left mouse button. Each inequality was displayed on the monitor until the participant provided the response; immediately after, a new inequality was presented. For each inequality, the four one-digit numbers and the three operators (+ or −) in the arithmetic operation were generated randomly, while the comparison target was generated as a random integer in the range r−3 to r+3, where r represented the correct result of the arithmetic operation. This small range disallowed a comparison target very distant from the possible result, thus precluding trivial solutions. Participants were instructed to answer as correctly and quickly as possible; speeding up the response was also encouraged by the appearance of the time left whenever a new inequality was presented. Response times (i.e. the time from the appearance of a new screen to item selection by mouse clicking) were collected for each subject.

This task included three attentional components: visual (to read numbers and symbols on the screen), computational (to solve the arithmetic operation) and motor (to move and click the mouse for selecting the response).

The mental task (Figure 1) consisted in solving the inequalities displayed on the monitor, without providing any explicit response. Therefore, this task did not require any hand and finger movement, i.e., it included the visual and computational attentional components, but excluded the motor one. The inequalities appeared on the monitor without the items for response selection and succeeded one another every 5 seconds. The time left was updated at each new presentation, in this case too. The numbers and operators in the arithmetic operation, and the comparison target were generated randomly as in the complete task. Participants were instructed to solve the inequalities as correctly and quickly as possible, even if their responses were not collected.

The reading task (Figure 1) was structured as the mental task (inequalities appeared without the response items every 5 seconds), but participants were instructed to just mentally read the numbers presented on the screen, without performing the arithmetic operation and solving the inequalities. Hence, both the computational and motor components were removed and only the visual attentional component was retained.

The finger movement task (Figure 1) consisted in displaying only the three response items (while the arithmetic operation and inequality were not displayed) and requiring the participants to randomly select one item with the mouse approximately every 5–10 seconds. Specifically, subjects were shown the approximate rate of item selection via a short demo by the operator (G.R) (i.e. they did not receive any instruction to count during the finger movement). After participant's selection, the screen was refreshed displaying the three items again for a new selection and updating the time left. Response times (i.e. the time from refreshing of the screen to item selection by mouse clicking) were collected for each subject.

This task excluded the visual and computational components associated with reading the inequalities and solving them, and retained only the visually-guided motor component.

During the two 5-minute relaxation phases, preceding (R1) and following (R2) each task, the same grey screen with the word “RELAX” was continuously displayed, and participants were instructed to relax during such phases maintaining the eyes open.

The four 15-minute experimental sessions were performed consecutively, separated one another by a 10-minute break; the order of the tasks was randomized across participants. During each experimental session, participants were asked to reduce body and head movements at minimum (except hand and finger movement for mouse use when required for task execution), and not to speak. During the 10-minute breaks between sessions, participants were allowed to speak and move on the seat and received the instructions for execution of the following task.

At the end of the four experimental sessions, each participant was interrogated about execution of the mental and reading tasks in order to ensure that he/she had understood correctly the different duty assigned in each of these two tasks and had not performed arithmetic computation during the reading task (in such case, the recording would have been rejected). Each participant confirmed to have performed correctly the two tasks (no recording was rejected). Two-third of them reported to have missed to finish solving inequalities during some trials of the mental task.

After collection of all recordings, we computed the response time averaged across all subjects in the complete task (mean = 6.6 s, Standard Deviation, SD = 3 s, median = 5.9 s) and in the finger task (mean = 4.97 s, SD = 2.9 s, median = 4.6 s) (see Section 4.1 for comments on this).

### 2.3. EEG and ECG recordings

EEG and ECG were measured simultaneously during the experimental sessions through a Neurowave System (Khymeia, Italy). The EEG signals were recorded using an elastic cap with 13 Ag/AgCl scalp electrodes. For the specific hypotheses of the present study, we focused on antero-central electrodes (F3, F4, T7, C3, Cz, C4, T8) and on posterior electrodes (PO7, PO3, PO8, PO4, O1, O2). Indeed, these electrodes cover regions linked to different information processing: motor coordination and execution for the more fronto-central regions [21] and visuo-spatial processing for the parieto-occipital regions [22],[23]. Therefore, this electrode placement was apt to investigate possible differential effects elicited over these regions by the different examined attentional components. Electrodes were referenced to the right earlobe, and the ground electrode was located on the forehead. One ECG lead was recorded by two Ag/AgCl electrodes attached just below the right and left collarbones. All electrode impedances was kept below 5 kΩ.

During each experimental session, the EEG and ECG data were digitized in continuous recording mode for 15 minutes at a sample frequency of 128 Hz and 16 bit resolution, and with the inclusion of a hardware notch filter eliminating line noise at 50 Hz. Thus, for each participant, four recordings lasting 15 minutes each were produced, relative to the four different tasks (overall 120 recordings were acquired). Furthermore, the 15-min ECG signal of each recording was off-line processed via the Neurowave software for automatic recognition of the R peaks and formation of the time series containing the RR intervals (i.e. intervals between two consecutive peaks), namely the tachogram. Finally, for each participant and each experimental session, the thirteen EEG signals and the tachogram were exported in a Matlab-compatible format for further analysis.

### 2.4. EEG processing

Processing of the EEG signals of each recording was performed using Matlab (R2016a, The MathWorks Inc., Natick MA). First, the EEG signals were high-pass filtered at 0.75 Hz to eliminate the DC offset and slow drifts. Then, for each participant and each experimental session (complete, mental, reading, finger-movement), the following processing steps were applied to the filtered EEG signals.

#### 2.4.1. Independent Component Analysis (ICA) and artifact removal

The filtered 15-minute EEG signals were decomposed into Independent Components (ICs) using the extended Infomax algorithm [24],[25] implemented in the open source EEGLAB Matlab toolbox (https://sccn.ucsd.edu/eeglab/index.php). Independent Component Analysis (ICA) is a widely-used statistical technique to find linear projections of the EEG data that are maximally independent, via minimization of their mutual information. Specifically, ICA finds an unmixing matrix (W) which, when multiplied by the EEG data, provides maximally independent signals (or components, the ICs) whose mixture have been recorded at the scalp. The inverse of the unmixing matrix (A = W^−1^, called mixing matrix) back-projects the ICs into the EEG scalp data. The separated ICs may represent both artefactual signals having non-cortical origin (e.g., potentials produced by eyeball movements, heart contraction, skeletal muscular activity, line noise artifacts etc.), and individual brain-origin signals compatible with synchronized activity within connected patches of the cortex [26]. The ability of ICA to separate non-cortical artefactual components from brain-origin components has made ICA a valuable tool for artifact removal from EEG data [26].

In this study, the unmixing matrix W was attained for the thirteen 15-minute EEG signals (via EEGLAB toolbox); then, the 15-minute temporal pattern of the thirteen estimated ICs, their Power Spectral Density (PSD), and their scalp maps were computed (outside EEGLAB toolbox). The scalp map of an IC shows the projection weights from the IC to each electrode location, i.e. the weights of the corresponding column of the mixing matrix A. For ICs' PSD computation, we used the same method and parameters adopted for the EEG signals (see below). By visual inspection of ICs' temporal pattern, PSD and scalp maps, we identified and rejected non-cortical ICs related to blinking, lateral eye-movements, heartbeat, muscular activity, and other clear artifacts. This procedure resulted in an average of 3.9 (SD = 0.83) independent components being rejected across all participants and experimental sessions. Artifact-cleaned EEG data were then reconstructed by back-projecting the remaining set of non-artefactual ICs.

Besides using ICA to remove non-brain source processes, here IC decomposition was used also to identify possibly meaningful brain-origin components by evaluating which of these components contributed most strongly to alpha power in the data during the individual tasks (see below for the detailed procedure). In particular, we aimed to assess whether independent components exhibited alpha power modulation more sensitive to the different tasks than undecomposed EEG signals.

#### 2.4.2. Alpha power computation over regions of interest

The artifact-cleaned EEG signals were subdivided into three parts of 5 minutes each, corresponding to the three phases of the session (R1, T, R2). The PSD of each channel over each phase was obtained by applying the Welch's periodogram method, by using a Hamming window of 5 seconds at 50% overlap, zeropadded to 10 s to obtain 0.1 Hz frequency resolution. Then, for our purposes, we topographically aggregated the channels into two regions of interest: a region (Fronto-Central-Temporal, FCT region) including the antero-central channels (F3, F4, T7, C3, Cz, C4, T8) and a region (Parieto-Occipital, PO region) including posterior channels (PO7, PO3, PO8, PO4, O1, O2). The mean PSD over the FCT and PO regions were computed by averaging the PSD across the corresponding channels, separately for each phase R1, T, R2. Then, the power in the alpha band 8-14 Hz was computed over each region of interest and for each phase R1, T, R2. For each region, the alpha power in the R1 phase was used as reference and the alpha power value in each phase was normalized with respect to this reference value. Normalization relative to the initial relaxation phase of each session was done to rule out possible confounding effect of participant's fatigue due to the execution of previous sessions, and to focus only on the changes induced by the specific task.

#### 2.4.3. IC contributions to the alpha power

In order to derive cues on independent processes mainly responsible for the alpha power observed over the two scalp regions, we computed the alpha power ascribed to each (non-artefactual) independent component. To this aim, the single (non-artefactual) IC was back-projected into the scalp channels, and the alpha power over the FCT and PO regions along the entire session duration was computed considering only the single back-projected component. This procedure was repeated for each IC, and the ICs were ranked based on the value of the alpha power they provided, separately for the FCT and PO regions. Next, within each ranking, we considered the two IC components that provided the highest contribution to the alpha power in the FCT ranking (1^IC-FCT, 2^IC-FCT) and in the PO ranking (1^IC-PO, 2^IC-PO). Then, the alpha power of each of these components was computed separately in each phase (R1, T, R2) of the experimental session and normalized with respect to the R1 value used as a reference.

Via this analysis, we aimed to discover individual components mostly accounting for the alpha power observed over the two scalp regions, which may be related to functionally distinct brain processes, and to assess whether these components were more distinctive of specific attentional aspects than undecomposed EEG signals. In perspective, these components may be exploited to drive neuroengineering applications (such as in neuro-ergonomic studies, BCI applications, and so on) without the need to use high-density EEG.

### 2.5. HRV analysis

For each participant and each experimental session, the following steps were applied to the RR interval time series to derive indexes of HRV.

First, the time series of the RR intervals was processed for recognition and correction of possible artifacts due to participant's movement, equipment failure or others. To this aim, we utilized the open source software ARTiiFACT (http://www.artiifact.de/) [27], that allows detection and treatment of artifacts in the tachogram. Specifically, within ARTiiFACT, the tachogram was submitted to the artifact detection algorithm proposed by Bernston et al. [28]. This is a widely used method for automatic artifact recognition; differently from other methods that detect artifacts as outliers that deviate from the mean/median more than a given threshold, the Berntson's method is based on the distribution of the differences in the RR intervals of the individual subject. Overall, in our RR time series, a limited number of artifacts was detected: 66 out of the 120 examined tachograms had no artifact; 42 tachograms had a percentage of detected artifacts below 1% (mean = 0.3%, SD = 0.25%); 9 tachograms had a parcentage of detected artifacts between 1% and 3% (mean = 1.64%, SD = 0.36%); the remaining 3 tachograms had a parcentage of detected artifacts ∼4.1%, ∼4.9%, and ∼7%. The detected artifacts in each tachogram were replaced using linear interpolation, within ARTiiFACT software.

The artifact-processed tachogram was then elaborated using Matlab. First, it was resampled at 10 Hz using linear interpolation and then low-pass filtered with a cut-off frequency of 0.5 Hz. The interpolated and filtered tachogram was subdivided into three portions each covering 5 minutes, corresponding to the three phases (R1, T, R2) of the experimental session. The PSD of each 5-minute portion was estimated via the periodogram Welch's method using a Hamming window of 100 seconds at 50% overlap, zeropadded to 500 s to obtain 0.002 Hz frequency resolution. The power in the low frequency band (0.04–0.15 Hz, LF power) and in the high frequency band (0.15–0.4 Hz, HF power) was then computed for each phase (R1, T, R2), and the ratio between the LF power and the HF power (LF/HF ratio) was derived too. Likewise the alpha power, the three indexes of HRV (LF power, HF power, LF/HF ratio) were normalized to the corresponding value in the R1 phase considered as the reference value.

### 2.6. Statistical analyses

Graphpad software (https://www.graphpad.com/) was used for statistical analyses. Statistical analyses were designed in order to:

a) Compare the normalized alpha power changes induced during the complete task with those induced during the other tasks, over each region of interest (FCT and PO) both in the measurement space and in the component (IC) space. Our hypothesis was that regional alpha power might be modulated not only by the number but also by the nature of the involved attentional components, linked to the functional role of the specific region.

b) To compare the normalized changes in the HRV indexes induced during the complete task with those induced during the other tasks. Our hypothesis was that HRV indexes could also depend on the amount of attentional engagement.

c) To explore possible correlation between changes in alpha power and changes in HRV indexes during the tasks.

Hence, the variables under statistical analyses were the normalized alpha powers and the normalized indexes of HRV, during each of the four tasks. Test for Gaussianity of each variable was performed using the omnibus K2 test. The hypothesis of Gaussianity was acceptable for each variable but the normalized LF/HF in the complete and mental tasks (*p* < 0.05). Therefore, the normalized LF/HF index in all sessions was square-root transformed to make it Gaussian in all sessions for parametric comparison.

Repeated measures ANOVA with Greenhouse-Geisser correction for violation of sphericity were used (uncorrected degrees of freedom and corrected probability levels are reported). One-sample *t* tests and post-hoc paired *t* tests were Bonferroni corrected for multiple comparisons. Correlation was tested using Pearson's correlation coefficient, with Bonferroni correction for multiple comparisons.

## 3. Results

### 3.1. Effect of tasks on alpha power

**Figure 2.**
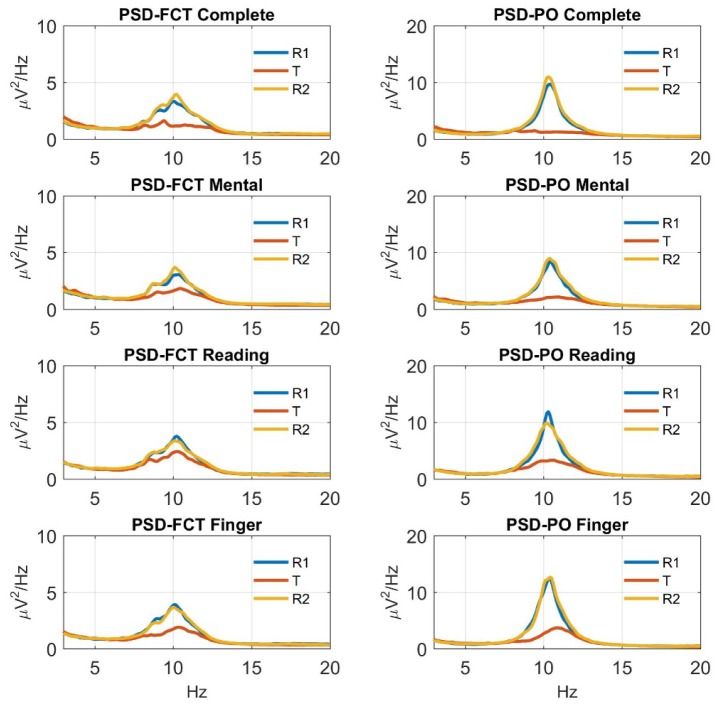
Power spectrum density (PSD) over the FCT region and the PO region computed during each phase (initial relaxation = R1; central task = T; final relaxation = R2) of the four experimental sessions (involving respectively the complete task, the mental task, the reading task, the finger movement task), averaged across the subjects. For each subject, the PSD over the FCT region and PO region were obtained by averaging the PSD of the channels F3, F4, T7, C3, Cz, C4, T8 and of the channels PO3, PO4, PO7, PO8, O1, O2, respectively. A smaller scale along the y-axis was adopted for the PSD over the FCT region in order to allow better appreciation of its modulation across the three phases of each session. The two relaxation phases of each session were characterized by a peak of the power spectrum in the alpha band, with larger values over the PO region. Each task phase was characterized by a decrease of the power spectrum in the alpha band, more consistent over the PO region.

Figure 2 displays the mean PSD over the FCT and PO regions computed during each phase (R1, T, R2) of the four experimental sessions (complete, mental, reading, finger movement), averaged across participants. First, we can observe that during each initial relaxation phase (R1), the PSD exhibited a peak in the alpha band (8–14 Hz), assuming particularly large values over the PO region (note the different scales used along the y-axis for the two regions). Second, during each task phase (T), the amplitude of the power spectrum in the alpha band showed a dramatic decrease over the PO region and a smaller decrease over the FCT region, compared to the corresponding R1 phase. Finally, during each final relaxation phase (R2), the power spectrum in the alpha band increased again, recovering or slightly overcoming the values in the corresponding R1 phase. These results are in line with the common finding that alpha oscillations are more evident during relaxation and are more prominent in the posterior regions.

**Figure 3.**
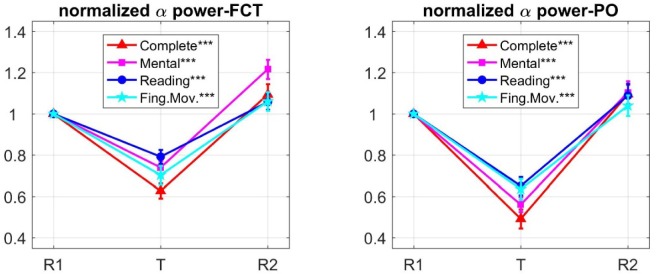
Mean ± standard error (SE, vertical bars) across subjects of the normalized alpha power during each phase (R1, T, R2) of the four experimental sessions, reported separately for the FCT region and the PO region. Values at R1 coincide with 1 since normalization in each phase of the session was done relative to the alpha power during the corresponding R1 phase (reference phase). In both regions, each task execution (T) induced a significant decrease in alpha power relative to the reference value (multiple one-sample t-tests); asterisks in the legend denote the level of significance (***p < 0.001, **p < 0.01, *p < 0.05; *p*-values were Bonferroni-corrected for four comparisons). However, the effect of the task type appeared to be non-uniform across the two regions, the different tasks modulating differently the alpha power (the four points in T had a different order in the two plots; see also Table 2).

Figure 3 plots the mean (± SE, Standard Error) across all participants of the normalized alpha power in the three phases of each experimental session, separately for the FCT region and the PO region. The alpha power exhibited a fall in all task phases relative to the corresponding R1 phase, assuming values in the range 60% to 80% of the reference value in the FCT region and in the range 45% to 65% in the PO region. Multiple one-sample t tests, applied separately for each region, confirmed that normalized alpha power significantly deviated from the reference value (1) during each task (see Figure 3). During R2, the alpha power recovered the reference value or slightly increased above it (see section Discussion for comments on alpha power rebound in R2). Interestingly, looking at the values in phase T, it is suggested that the different tasks could modulate differently the alpha power in the two regions: indeed, in the PO region the effect of the complete task appeared closer to that of the mental task, whereas in the FCT region the effect of the complete task was closer to that of the finger movement task.

To investigate the effect of the task's type over each region, a repeated measures one-way ANOVA with task type (complete, mental, reading, finger movement) as within-subject factor was conducted on the normalized alpha powers during the task phases. Results indicated a significant main effect of task type over both the FCT region (F_3,87_ = 7.810, *p* < 0.001) and the PO region (F_3,87_ = 6.734; *p* = 0.002). Post-hoc paired t-tests were performed comparing the complete task with each of the other tasks, in each region (Table 1). In the FCT region, the complete task came out to be significant different from the reading and mental tasks but not from the finger task, while in the PO region the complete task was significant different from the reading and finger tasks but not from the mental task. Hence, the different tasks impacted unevenly over the two regions, the complete and finger tasks producing similar alpha power modulation over the fronto-central but not the posterior region, whereas the complete and mental task producing similar effects over the posterior but not the fronto-central region. These results suggested that distinct and differently regulated processes contributed to the alpha oscillations over the two regions. To gain cues on this, we performed an analysis at the level of independent components.

**Table 1.** Results of the post-hoc paired t-tests comparing the complete task against each of the other tasks over each region (FCT and PO) separately (reported *p*-values are Bonferroni corrected for three comparisons).

	FCT region	PO region
Complete task vs Mental task	**p = 0.0012	*p* = 0.07
Complete task vs Reading task	***p < 0.001	***p < 0.001
Complete task vs Finger task	*p* = 0.1	**p = 0.006

**Figure 4.**
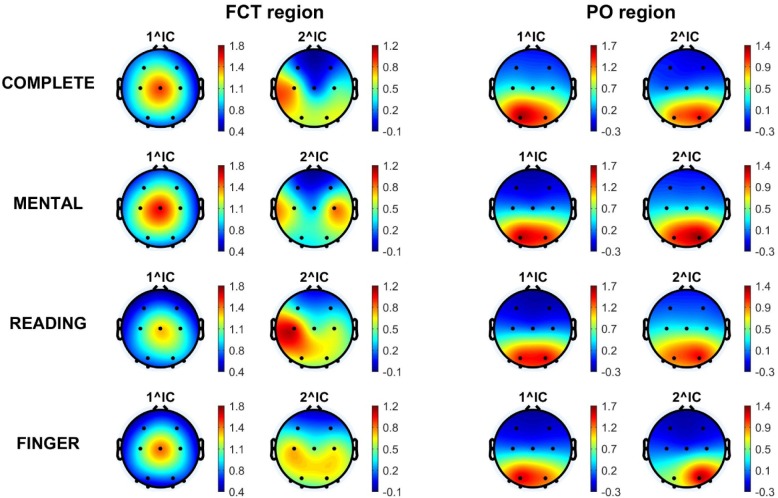
Mean scalp maps averaged across participants showing the projection weights of the two ICs that more strongly contributed to alpha power over the FCT region (the two columns on the left) and over the PO region (the two columns on the right). Each row refers to a specific experimental session. On average, the two ICs explained more than 70% of alpha power over each region respectively, with the first one accounting for about the 50%.

The two ICs (1^IC and 2^IC) that mostly explained alpha power over each region were identified per each participant and experimental session (see Material and Methods). Their topographical maps, averaged across participants, are shown in Figure 4. Overall, each of the identified components exhibited averaged scalp distributions quite similar across the four experimental sessions (see the maps along each column in Figure 4). Importantly, the identified ICs exhibited different scalp distributions depending on the considered region. Notably, among the 480 identified ICs (2 ICs x 2 regions x 4 experimental sessions x 30 participants), the same IC was identified for both the FCT and PO regions (either at the first or second position in the rank) limitedly to 9% of cases. Accordingly, alpha activity over the regions FCT and PO may be mostly ascribed to independent brain processes characterized by different scalp topographies. As to the FCT region, the first contributing IC exhibited a central maximum and symmetrical patterns towards frontal and posterior areas; the second contributing IC had lateral centro-parietal maxima and extended towards posterior-parietal areas. As to the PO region, both the two identified ICs had scalp distribution sharply confined over the posterior areas.

**Figure 5.**
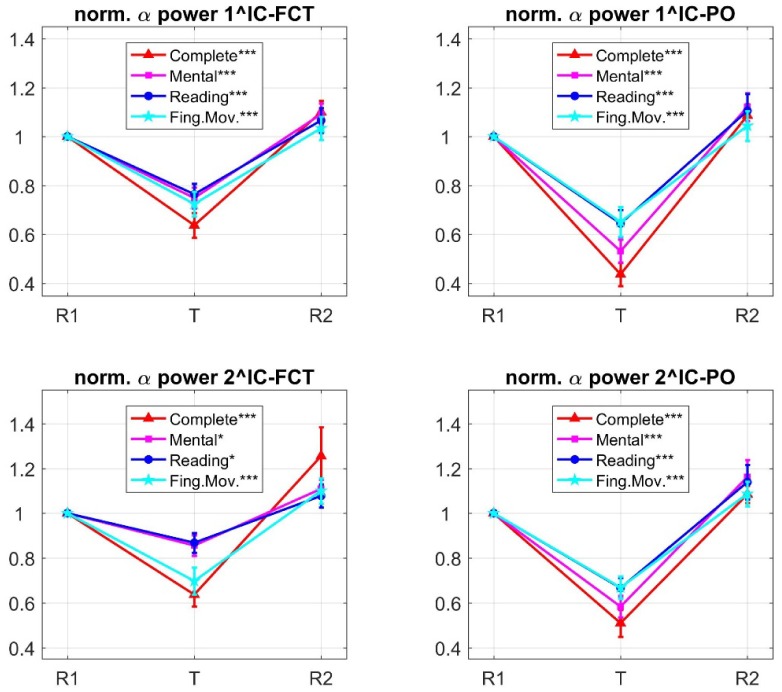
Mean ± standard error (SE, vertical bars) across subjects of the normalized alpha power during each phase (R1, T, R2) of the four experimental sessions, computed on the 1^ and 2^ ICs contributing to FCT alpha power (1^IC-FCT, 2^IC-FCT) and on the 1^ and 2^ ICs contributing to PO alpha power (1^IC-PO, 2^IC-PO). In both regions, each task execution (T) induced a significant decrease in alpha power relative to the reference value (multiple one-sample t-tests); asterisks in the legend denote the level of significance (***p < 0.001, **p <0 .01, *p < 0.05; p-values were Bonferroni-corrected for four comparisons). The components 1^IC-FCT, 2^IC-FCT exhibited task-dependent alpha power modulation (phase T) in line with that observed at the FCT channels, i.e. the complete-task and the finger-task were associated with larger and closer alpha power decrease (especially in the 2^IC-FCT). The components 1^IC-PO and 2^IC-PO (right plots) exhibited task-dependent alpha power modulation consistent to that observed at the PO channels, i.e. the complete task and the mental task were associated with larger and more similar alpha power decrease.

The identified ICs were then analyzed in terms of normalized alpha power changes during each task phase (see Material and Methods). Values (mean ± SE) averaged across participants are plotted in Figure 5. For each IC, multiple one-sample t-tests confirmed significant deviation of normalized alpha power from 1 during each tasks (see results in Figure 5). Plots in Figure 5 suggest that each IC exhibited a pattern of task-dependent alpha modulation similar to that observed at the corresponding channels level (Figure 3). As to the 1^IC-FCT and 2^IC-FCT, the finger task produced an alpha power decrease approaching that induced by the complete task (this is especially evident in the 2^IC-FCT). Conversely, as to the 1^IC-PO and 2^IC-PO, the mental task produced the effect closest to the complete task. Repeated measures one-way ANOVA with task type as within-subject factor was applied to normalized alpha power of each IC (in the task phase), showing a main effect of task (1^IC-FCT: F_3,87_ = 3.124, *p* = 0.037; 2^IC-FCT: F_3,87_ = 5.278, *p* = 0.003; 1^IC-PO: F_3,87_ = 8.693, *p* < 0.001; 2^IC-PO: F_3,87_ = 3.951, *p* = 0.015). Post-hoc paired t tests (Table 2) proved that the 1^IC-FCT and especially the 2^IC-FCT emphasized the non-significant deviation between the complete and the finger task, maintaining a significant (or close to significance) difference between the complete task and the mental and reading tasks. On the other hand, 1^IC-PO and 2^IC-PO (Table 2) emphasized the non-significant difference between the complete and mental tasks, while presented significant difference of the complete task from the finger and reading tasks.

Overall, these results are suggestive of functionally distinct brain processes subtending alpha oscillations over more central regions and more posterior regions; in the former, alpha synchronization appears to be mainly reduced by motor and visuomotor attentional components, whereas in the latter alpha power seems to be mainly reduced by visual-computational attentional components.

**Table 2.** Results of the post-hoc paired t-tests performed on the normalized alpha powers of each examined independent component (1^IC-FCT, 2^IC-FCT, 1^IC-PO, 2^IC-PO); the tests compared the effect of the complete task against each of the other tasks, separately for each component (reported *p*-values are Bonferroni corrected for three comparisons).

	1^IC-FCT	2^IC-FCT	1^IC-PO	2^IC-PO
Complete task vs Mental task	*p* = 0.061	**p = 0.006	*p* = 0.13	*p* = 0.756
Complete task vs Reading task	*p = 0.035	**p = 0.005	***p < 0.001	*p = 0.024
Complete task vs Finger task	*p* = 0.3249	*p* > 0.999	***p < 0.001	*p = 0.014

### 3.2. Effect of tasks on HRV and correlation tests between HRV and alpha power

**Figure 6.**
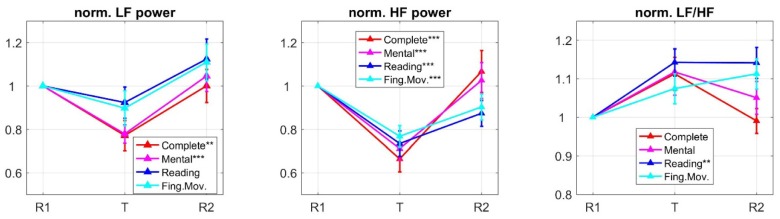
Mean ± standard error (SE, vertical bars) across subjects of the normalized HRV indexes (LF power, HF power and the ratio LF/HF) during each phase (R1, T, R2) of the four experimental sessions. The normalization was done relative to each corresponding R1 phase (producing values equal to 1 in R1). The values plotted in the rightmost plot refer to root-square transformation of the normalized LF/HF index, to make it Gaussian for parametric analyses. Overall, during each task execution HF power decreased more than LF power, and LF/HF ratio tended to increase.

Figure 6 shows the mean (± SE) across participants of the HRV indexes (LF power, HF power, ratio LF/HF) in the three phases of each experimental session, normalized relative to the corresponding R1 phase. Moreover, the figure reports the results of multiple one-sample t-tests performed for each index during the tasks. During task execution HRV tended to reduce especially in the HF band, while the LF power decreased to a lower extent. This was reflected in a tendency of LF/HF ratio to increase.

Repeated measures one-way ANOVA with task type as within-subject factor were applied to each HRV index during the task phases, revealing no main effect of task on any index (LF power: F3, 87 = 1.589, *p* = 0.206; HF power: F3, 87 = 0.924, *p* = 0.419; LF/HF ratio F3, 87 = 0.478, *p* = 0.687). Hence, although the mental and complete tasks seemed to produce larger effects compared to the reading and finger tasks both in the LF and HF bands (Figure 6), there were not significant differences across the tasks. We can conclude that HRV indexes are less sensitive to different task types involving different attentional components, compared to EEG alpha power indexes.

Finally, we tested the correlation across all subjects between the HRV indexes (LF index, HF index) and each of the alpha power indexes (normalized alpha power of the two scalp regions and normalized alpha power of the four selected independent components), separately during each task; that is, six Pearson's tests were performed for each HRV index during each task, with Bonferroni corrected *p*-value. No significant correlation was found with alpha power for either LF index (minimum corrected *p*-value = 0.14) or HF index (minimum corrected *p*-value = 0.23), in any task.

## 4. Discussion

In this study, we investigated the differential effect of tasks involving different combinations of attentional components (visual, computational, motor) on both brain activity and autonomic nervous activity. The effect on brain activity was analyzed through quantification of task-related EEG alpha-power changes in two regions of interest over the scalp (a fronto-central region and a posterior region). Moreover, independent component decomposition of EEG data was applied in order to get cues on possibly distinct brain processes contributing to alpha-power modulation and to extract relevant individual components for future possible uses in neuro-engineering applications. The effects on autonomic nervous activity were analyzed via HRV indexes (the power of the tachogram spectrum in the LF and HF bands) and quantification of their task-related changes. Finally, the correlation between alpha power measurements and HRV indexes during the attentional tasks was tested.

### 4.1. Task-related modulation of alpha power

The first general result of our study is that a significant alpha power decrease, relative to the baseline (the pre-task relaxation phase), was observed (Figure 3) over both the front-central and the parieto-occipital regions during each task. Alpha power increase was never observed on either scalp regions during the examined tasks, in line with previous studies showing that increase in alpha activity occurs when distracting or confounding information must be actively blocked (e.g., when attended one side of the space while distracting information on the other side must be ignored), a condition not occurring in our tasks. The significant alpha power decrease observed over both scalp regions suggests that the influence of the attentional components involved in each task was widely distributed, each task requiring the co-participation of several areas and brain processes. This broad alpha power suppression observed in all examined tasks is also in line with previous findings. Previous EEG studies involving mental arithmetic (specifically addition and subtraction) found alpha power decrease especially in parietoccipital regions but also in frontocentral regions [7]–[9],[15]. Interestingly, widespread alpha power suppression occurred in relatively complex tasks (e.g., addition of two-digits numbers, operations with more than two operands, operations involving subtraction), similar to the operations used in this study, while it was less evident in simpler task (e.g., 2 + 3). Fernandez et al. [16] during a task involving the reading of multiple digit numbers observed a significant alpha power decrease in all 19 EEG channels (located according to the traditional 10/20 system). In motor tasks, alpha power suppression was not confined over motor areas [29],[30], especially when also sensory information (e.g., tactile and/or visual) needs to be integrated, as actually occurred in our finger movement task.

Despite the generalized alpha power decrease over both regions during the tasks, a difference emerged between the two regions. This difference appears consistent with the main functional role of the underlying cortical areas. Precisely, in both regions the complete task (having the larger number of attentional components) tended to produce the largest alpha power suppression and the reading task (involving only visual component) the lowest alpha power suppression, while the other two tasks (the finger movement and mental tasks) were collocated approximately in between. However, while in the fronto-central region, the complete task presented larger (and significant) deviation from both the mental and reading tasks, it presented a smaller deviation (not reaching significance) from the finger task. On the other hand, while in the parieto-occipital region the complete task showed larger and significant deviation from both the finger and reading tasks, it showed a smaller deviation (not reaching significance) from the mental task. Overall, these results are suggestive of a graded relationship between the number of involved attentional components and extent of alpha suppression (the larger the number of attentional components the larger the suppression in alpha power). However, this graded relationship did not have a uniform pattern across brain regions, but it was fine-tuned based on the main functional role of the underlying cortical areas. These observations are in line with the hypothesis that alpha modulates attentional cortical resource allocation. Indeed, a task entailing a larger numbers of attentional components will engage several cortical areas, requiring disinhibition of more numerous and larger assemblies of neurons (so that they may become entailed in specific information processing) and thus in general will provide larger alpha power suppression. However, the involved attentional components will mainly affect the areas more strongly implicated in the corresponding information processing, and to a less extent more marginal areas. Indeed, recent EEG studies suggest that the posterior parietal and occipital regions are involved in visuo-spatial processing of stimuli [22], spatial representation of numbers [23] and complex (vs simple) arithmetic problems requiring procedural strategies [7],[31]. Accordingly, in the present study alpha power over these areas appeared to be strongly affected by the visual and computational attentional components of the complete and mental tasks, while the reading task and the finger movement task required relatively lower recruitment of these areas. Conversely, fronto-central areas are strongly implicated in sensory-motor integration and motor execution, and therefore they were strongly entailed by the visuo-motor component of both the complete task and the finger task (while their recruitment in the reading and mental task was relatively lower).

Motivated by this interpretation, we extracted independent components that mainly contributed to EEG power over each region. Based on the previous results, we expected that the components mainly responsible for alpha power over each region were distinct and differently attention-modulated, possibly being related to brain processes that subserve distinct functions. Indeed, we found that the two main contributions to alpha power over the fronto-central channels and the parieto-occipital channels were provided by different independent components, having scalp topography and task-related power modulation (Figures 4 and 5) more compatible with sensory-motor and motor processes on one hand, and visuo-spatial-calculation processes on the other hand. Of course, association between scalp topographies and cerebral processes/functions remains at a speculative level here, due to the unfeasibility of applying algorithms that estimate (distributed or concentrated) cortical sources, because of the limited number of electrodes. However, we provide a cautious neurophysiological explanation of these analyzed independent components, which undoubtedly requires further investigation and evidence. As to the 1^IC-FCT, it could be related to a multisensory-motor process (localized in frontal or anterior-parietal cortex), supporting the integration of multisensory (tactile, visual, proprioceptive, etc.) information—but also endogenously generated information (e.g., computation result)—with motor behavior and response. Indeed, as to its task-related alpha power modulation, the largest alpha power suppression was observed in the complete task, where all the previous information must be integrated, and was found to exhibit the lowest deviation from the finger movement task where still multisensory and motor information needed to be integrated. The 2^IC-FCT might be implicated in final motor execution, located in the motor cortex; indeed its alpha power suppression was the largest in the complete and finger tasks and exhibited very similar value in these two cases. As concerning the two main components of PO alpha power (1^IC-PO and 2^IC-PO), they could be related to occipital and posterior-parietal processes that become triggered in visuo-spatial processing and in mathematical cognition; their alpha power suppression is most prominent (and not different) in the complete and mental tasks. The similarity of the averaged scalp maps of the two independent components 1^IC-PO and 2^IC-PO (Figure 4) deserves a clarification. Actually, for each single subject, these two components turned out to have scalp maps with a pretty clearly lateralized peak, located posteriorly on the left (over positions PO3, PO7, O1) for one component, and on the right (over position PO4, PO8, O2) for the other, each possibly reflecting (visuospatial/computational) processes relative to one hemisphere. However, there was not consistency across subjects and tasks concerning the rank in contributing to PO alpha power (1^ or 2^ position) and the left- or right-peaking of these two components: sometimes, the left- (right-) peaking component occurred to provide the first (second) contribution to PO alpha power and sometimes the vice-versa occurred. Therefore, when averaging their scalp maps across subjects, lateralization tends to disappear creating a pretty uniform (with just a small left or right bias) posterior distribution.

Two further points regarding alpha power changes obtained in our study deserve comments.

First, in the mental and reading tasks, there was a predefined time window (5 s) imposing the rate of screen refresh (0.2 Hz), i.e. the rate of trials, while in the complete and finger tasks a predefined time window was not imposed possibly slowing the average rate of trials (defined by the average response time, see Section 2.2.). This task difference might constitute a source of bias of alpha power, by reducing the level of stress and thus producing underestimation of alpha power decrease in the complete and finger tasks compared to the mental and reading tasks. While we cannot exclude this possibility, we are inclined to consider this effect insignificant. Indeed, the finger task turned out to be characterized by an average rate of trials close to 0.2 Hz (as the mental and reading tasks). Moreover, we have verified that in the finger task, there was not correlation between the rate of item selection (i.e. the rate of finger movement) and the alpha power decrease across the thirty participants (*p* > 0.39). For what concerns the complete task, the average rate of trials was effectively lower (average response time = 6.6 s); this might have introduced a positive bias (reduced alpha decrease) compared to the mental task in particular, as both tasks required to solve inequalities. However, in the mental task, each assigned time window was only devoted to solve the inequality (without spending time for identifying the item matching the solution, moving the mouse over it, clicking the mouse); moreover, most of participants admitted to have not finished solving inequalities in some trials of the mental task. Accordingly, we reasonably claim that the mental task did not involve additional workload and attentional effort in inequality solving compared to the complete task.

A second point concerns the restriction of our analysis to alpha power changes during the tasks, whereas also analysis of alpha rebound in R2 (relax post-task) might potentially provide further information about the involved attentional processes. Actually, statistical analyses applied to normalized alpha powers in the R2 phase failed to identify significant deviation from R1 phase (indeed, significance occurred only for R2 alpha rebound in the FCT region as to the mental task) and failed to identify differences across tasks. Further studies are necessary to assess whether the absence of task effect in R2 at scalp level is a genuine result, or rather a task effect in R2 occurs at cortical level and is masked by the volume conduction phenomenon and low electrode spatial resolution (maybe the task effect at phase R2 being of lower magnitude compared to the task effect at phase T).

Overall, we posit that the present study not only provide an incremental contribution to our knowledge of the electroencephalographic features of attention, and of their modulation by the number and nature of involved attentional components, but may have practical implications, too. Indeed, the obtained results suggest that even the use of a limited number of electrodes, as adopted in clinical settings or in neuro-engineering applications, if combined with independent component analysis, may allow the extraction of independent signals that better characterize either visual-computational aspects of attention or motor aspects of attention. This can open interesting possibilities in future applications where extracting relevant attentional features (more motor-related or cognitive-related) from a simple-to-use EEG system may have significant practical value, such as in neuroergonomics applications (e.g., in work and driving settings), in BCI applications, but also in clinical applications e.g. for monitoring attentional level in subjects with attention-deficit disorders.

### 4.2. Task-related modulation of HRV indexes

Our results concerning HRV indexes indicated a general decrease in HRV during task execution, characterized by a more relevant decrease in the HF band than the LF band and a consequent unbalancing towards sympathetic activity relative to parasympathetic activity (as indicated by the tendency of LF/HF ratio to increase). Reduction in HRV (especially in vagally-mediated HRV) during attentional demanding tasks has been documented in literature [9],[13],[20],[32],[33]. Furthermore, some of these studies also showed sensitivity of HRV indexes to different levels of attention or workload. In our study, although tasks could induce significant changes in the HRV indexes compared to baseline, none of the examined indexes exhibited significant differences across the tasks (see section Results). However, in the previous studies, participants performed each task for more than 10 minutes, while here each task lasted 5 minutes. A possibility is that changes in autonomic regulation of HRV might require more time to develop completely.

When correlation was tested between alpha power indexes and HRV indexes, no significant results was obtained. Investigating the relationship between oscillatory neural activity and HRV in relaxation and attention condition may contribute to disclose cortical processes influencing cardiovascular control in attentional states, and to improve our understanding on how our brain operates to react to attentional loads not only at central but also at peripheral level. Within this issue, some previous studies found significant correlation between EEG oscillatory activity and indexes of cardiac autonomic activity both in attentional states [9],[34] and during relaxation [35],[36]. However, in most cases, only low magnitude of correlation (∼0.3, 0.4) was found. We claim that understanding the information dynamics between brain and heart will benefit from the use of more sophisticated methods of analysis, able to capture not only linear, but also non-linear associations, such as the recent emerging methods based on transfer entropy to investigate the causal relationship among temporal series [37].

Finally, other lines for future researches, aimed at complementing and enriching the present results, are shortly mentioned.

First, in the present study we limited our investigation to alpha oscillatory activity as the main general mechanism supporting attention-related shaping of cortical engagement/disengagement. In future, it will be important to associate measure of alpha band activity with measure of cortical oscillatory activity in other bands (e.g., gamma). Indeed, increase in alpha activity amplitude has been suggested to break ongoing gamma activity; therefore, investigation of the interplay between gamma and alpha activity and of how this interplay is modulated by different attention levels and different attentional components will further improve our understanding of the neural correlates of attention. However, a similar investigation will require higher electrode density and more sophisticated paradigms, for emergence of gamma rhythm.

Moreover, as previously mentioned, the present study used a limited numbers of electrodes. This precluded both the possibility of parceling the scalp into several regions of interest, and more importantly the possibility of estimating cortical source localization via distributed source models or via equivalent dipole fitting. The interesting and novel results that have emerged from this preliminary work provide motivation to deepen the underlying neural mechanisms via high-density EEG and algorithms of cortical source reconstruction. Moreover, via reconstruction of the activity in cerebral structures, the dynamic coupling between autonomic and cerebral signals can be investigated in a more reliable way.

## 5. Conclusions

In conclusion, this study examined the influence of different combinations of attentional components on EEG alpha oscillatory activity and indexes of heart rate variability. The results indicated that modulation of EEG alpha power over different regions is sensitive to the involved attentional components, being not only regulated by the coarse level of attentional demand, but also fine-tuned by the nature of the mechanisms recruited (visual, motor, cognitive) relative to the functional specification of the areas. These data provide further support to the role of alpha oscillatory activity as a neural mechanism subserving attention via allocation of cortical resources and routing of information processing in a finely and goal-oriented manner. Heart rate variability indexes appear less sensitive to different attentional components compared to alpha power, with the index of vagal tone (HF power) presenting larger modulation. Overall, the present results may have implications in applied settings such as human-factor engineering, neuroergonomics, brain-computer interfaces, but also in clinical practice and in neuroscience research investigating electroencephalographic and autonomic correlates of attention disorders.
